# Growing Embossed Nanostructures of Polymer Brushes on Wet-Etched Silicon Templated via Block Copolymers

**DOI:** 10.1038/srep20291

**Published:** 2016-02-04

**Authors:** Xiaobin Lu, Qin Yan, Yinzhou Ma, Xin Guo, Shou-Jun Xiao

**Affiliations:** 1 State Key Laboratory of Coordination Chemistry, Collaborative Innovation Center of Chemistry for Life Sciences, School of Chemistry and Chemical Engineering, Nanjing University, Nanjing 210093, Jiangsu, China

## Abstract

Block copolymer nanolithography has attracted enormous interest in chip technologies, such as integrated silicon chips and biochips, due to its large-scale and mass production of uniform patterns. We further modified this technology to grow embossed nanodots, nanorods, and nanofingerprints of polymer brushes on silicon from their corresponding wet-etched nanostructures covered with pendent SiH*x* (*X* = 1–3) species. Atomic force microscopy (AFM) was used to image the topomorphologies, and multiple transmission-reflection infrared spectroscopy (MTR-IR) was used to monitor the surface molecular films in each step for the sequential stepwise reactions. In addition, two layers of polymethacrylic acid (PMAA) brush nanodots were observed, which were attributed to the circumferential convergence growth and the diffusion-limited growth of the polymer brushes. The pH response of PMAA nanodots in the same region was investigated by AFM from pH 3.0 to 9.0.

One of the key achievements for many future applications of nanostructures is to precisely pattern nanoscale features on technologically relevant semiconductor surfaces, such as silicon and germanium, to improve the chip performance and invent new technologies whereas continuing to reduce the chip size. Recently, block copolymer lithography, an emerging nanolithography technique, has received considerable attention in academics and industry^1^,^2^,^3^,^4^,^5^,^6^. The most attractive advantage of block copolymer lithography over other physical nanolithographies, such as electron- or ion-beam lithography, scanning probe lithography, imprinting, and Dip-Pen nanolithography, is the mass and very-large-scale (up to an entire wafer size) production of densely packed nanostructures within a 100-nm scale at minimal expense and with easy handling. The physical techniques for nanolithography have the limitations of requiring expensive machines and having high cost, tedious operation, intensive labor, and chip production at a small scale of several millimeters. Block copolymers provide diverse shapes of nanostructures on the supporting semiconductor substrates, which are mainly silicon, including spheres, cylinders, lamellae, and interconnected networks, with fine tunability over the nanometer scale range of 10 to 100 nm, depending on their molecular weights, compositions, and environmental conditions^7^,^8^. The most intensively investigated application is the transfer of these nanostructural templates into silicon by dry etching — ion etching (purely physical) or plasma etching (purely chemical). This etching removes one component of the block copolymer and further etches into silicon to generate intaglio (or indented) nanostructures. The modification of the block copolymer nanolithography by wet (incubation) or dry (sputtering or evaporation) deposition of a metal or metal oxide onto the pre-designed patterns generated functional nanodot or nanowire arrays^9^,^10^,^11^. Versatile applications of block copolymer nanolithography have been reported in semiconductor technologies (e.g., bit patterned media^12^), sensors^13^, and biomedicine^14^.

Polymer brushes are densely packed polymer arrays with one end fixed to a surface^15^,^16^,^17^,^18^,^19^. There are two general methods to synthesize tethered polymer chains: physisorption and covalent attachment. In physisorption, the ionic Coulomb interaction is often the driving force for the adsorption of polymer species carrying opposite charges onto a suitable substrate. However, weak interactions between the substrate and polymer can lead to thermal and solvolytic instabilities^20^,^21^. In contrast, covalent grafting, particularly a living “grafting from” method, is well known for its excellent long-term stability and chemical resistance of polymer brushes under stringent environments^22^,^23^. In this technique, a surface-immobilized initiator starts the polymerization and extends to polymer brushes by the addition of monomers. As a result, polymer brushes with thicknesses of up to several hundreds of nanometers can be readily achieved. The practical applications of polymer brushes have attracted considerable interest in many scientific fields, such as biomaterials, biosensing, biochips, drug delivery, nanofluidic devices, organic electronic devices, and functional membranes for energy and environments^24^,^25^.

In humans, chip-body interactions are a specific application of Si-based electronic chips, which have inspired innovative studies on brain implant chips, neurochips, tissue chips, and organ chips^26^. These chips require a buffer layer between the living tissues and the chip body of silicon, which is one of the driving forces to integrate polymer brushes into nanopatterned silicon chips. Growing polymer brushes on micro- and nanopatterned silicon has been reported using colloid lithography at the micrometer scale and using electron-beam lithography at the nanometer scale in which the initiator monolayer was linked to silicon by surface silane chemistry^27^,^28^. Block copolymer lithography with wet etching offers an advantage for the mass and very-large-scale (up to an entire wafer size in tens of centimeters) production of chips in a general chemistry laboratory at minimal expense and with easy handling. Wet etching of silicon is one of the fundamental techniques of micro- and nanofabrication. Fortunately, wet etching not only produces nanopatterns but also provides surface reactive SiH*x* species, which can be used to covalently graft organic, polymer, and biomolecular ultrathin films via surface hydrosilylation chemistry^29^,^30^,^31^,^32^,^33^. The robust silicon−carbon bond interfaces with silicon and soft materials, generating hybrid devices, presenting great promise and appeal for new applications, such as molecular electronics, nano-electronics, silicon photonics, neurochips, and biochips. However, few studies have considered the use of block copolymer lithography for the direct nanopatterning of covalently bound molecular monolayers and polymer brushes^34^,^35^,^36^,^37^.

In this study, we endeavored to grow polymer brushes via surface-initiated atom transfer radical polymerization (SI-ATRP)^15^,^16^,^17^,^18^,^19^ on block copolymer templated nanostructures of silicon. Briefly, a block copolymer film of PS-*b*-P4VP micelles was coated on silicon as the template, where nanodots or nanorods of P4VP aggregates, depending on their molecular weights, were generated from the PS matrix^7^,^8^,^9^,^10^. Controlling the wet etching of silicon with dilute aqueous HF to an appropriate depth, e.g., 5 to 20 nm, is critical to produce homogeneous nanopits or nanoslits and to further convert these intaglio nanostructures into embossed structures of polymer brushes. Overetching will produce irregular pattern shapes, whereas underetching will not provide a sufficient amount of surface reactive SiH*x* species. The polymer brush sizes regarding height and volume, which are limited to a maximum of a few hundred nanometers, can be tuned by the polymerization conditions, such as immersion time and catalyst and monomer concentrations. After the application of hydrosilylation chemistry with vinyl-ended molecules to form the surface initiator monolayers, SI-ATRP was conducted on the SiH*x*-covered nanostructures to form the embossed nanodots or nanorods of polymer brushes using the “grafting-from” approach. Our efforts using the same etching approach to the annealed PS-*b*-P2VP nanofingerprints to extend to nanowires of polymer brushes, such as fingerprints, were unsuccessful. An alternative approach by wet etching platinum (Pt) dotted nanofingerprints derived from PS-*b*-P2VP nanofingerprints^9^,^10^, followed by surface hydrosilylation and SI-ATRP provided satisfactory embossed nanofingerprints of polymer brushes. These stepwise surface reactions were monitored with AFM for topomorphological changes and with multiple transmission–reflection infrared spectroscopy (MTR-IR)^38^,^39^,^40^, except for the SiH*x* species, for molecular characterization.

## Results and Discussion

### Inversion of the intaglio nanopits etched from PS-*b*-P4VP templates to nanodots by growing PMAA brushes

**Figure 1:**
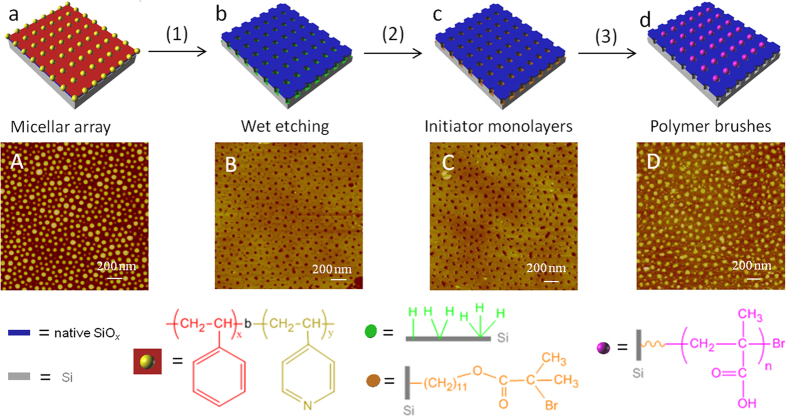
Schematic procedure for the fabrication of embossed polymer brush nanodots and their corresponding AFM images. The schematic cartoons and their corresponding AFM images are shown as follows: (**a** and **A**), diblock copolymer template of PS-*b*-P4VP; (**b** and **B**), etched nanopits; (**c** and **C**), initiator monolayer covalently bound to nanopits; (**d** and **D**), PMAA brush nanodots. The three steps are as follows: (1) wet etching, (2) initiator grafting, and (3) SI-ATRP. The physical meanings of the schematic labels are illustrated at the bottom.

Fig. 1 presents the schematic fabrication procedure with cartoons and their corresponding real AFM images in each step. Briefly, a micellar solution of PS-*b*-P4VP in toluene (0.5% w/w) was prepared by stirring at 40 °C for 12 h. Then, the solution was spin-coated on a 20 × 20 or 10 × 10 mm^2^ silicon chip, depending on the application, to form a 1-μm-thick film. The block copolymer of PS-*b*-P4VP with a total molecular weight of ca. 136,000 g/mol (Mn = 109,000–27,000 g/mol) and a polydispersity of 1.1 adopted a quasi-hexagonal close-packed array of vertical P4VP cylinders^10^ surrounded by a PS corona (cartoon in Fig. 1a and AFM image in Fig. 1A). Wet dilute HF etching generated a SiH*x* pendant nanopit array (cartoon in Fig. 1b and AFM image in Fig. 1B) after relieving the PS-*b*-P4VP film. During the wet chemical etching process, the hydrated HF molecules penetrated the hydrophilic P4VP cylinders and protonated the pyridyl groups to form poly(pyridinium)fluoride. Through the protonated nanochannels, hydrated HF molecules completely etched the native oxide silica layer (approximately 1.0 nm) and continued to etch silicon slowly in an anisotropic manner into intaglio nanopits, where pendant SiH*x* groups were generated inside the intaglio surfaces. After etching, the block copolymer film was removed via 10–20 min of ultrasonication in neat toluene and trichloromethane sequentially. Etching exclusively occurred beneath the protonated P4VP cores of the polymer micelles, mirroring its original P4VP dot array in Fig. 1A, and the PS matrix in the copolymer film protected its underlying native silica, which remained intact and flat on the top surface. Because the etching is spatially selective, the interior chemical functionalities inside the nanopits differ from those on the flat top surface. Therefore, parallel functionalization strategies in which only the nanopit interiors were modified separately from their surrounding top surface areas were feasible, allowing the simultaneous generation of more sophisticated and highly ordered nanostructures.

Surface hydrosilylation was executed on a freshly etched chip (10 × 10 or 20 × 20 mm^2^) with 10-undecen-1-yl-2-bromo-2-methylpropionate (10 mL) covering the chip surface in a vessel under microwave irradiation (125 °C for 30 min)^41^,^42^, generating a self-assembled monolayer with an end initiator of α-bromoisobutyrate (cartoon in Fig. 1c and AFM image in Fig. 1C). The AFM image of Fig. 1C does not show a large difference from the freshly etched nanopits in Fig. 1B because the initiator monolayer is theoretically only 1.2 nm high. Previously, we applied two-step reactions to graft the surface initiator of α-bromoisobutyrate onto silicon^43^,^44^. Briefly, ω-undecenyl alcohol was first grafted by surface hydrosilylation; then, the surface initiator was attached through the acylation of α-bromoisobutyryl bromide. However, the release of HBr during acylation resulted in an acidic environment, which, combined with a long incubation time over 12 h, presented harsh reaction conditions, which occasionally damaged the surface nanostructures. In addition, the two-step reaction yield is lower than that of a one-step reaction; thus, the surface density of initiators is not sufficiently high to initiate ATRP. In this study, we first synthesized 10-undecen-1-yl-2-bromo-2-methylpropionate^45^,^46^ in bulk and then executed the surface hydrosilylation reaction in one step to introduce the initiator monolayer of α-bromoisobutyrate within the SiH*x* pendant nanopits.

The surface coverage of α-bromoisobutyrate, controlled by the surface hydrosilylation chemistry, can be roughly screened with MTR-IR on a 20 × 20 mm^2^ chip for further ATRP. After rinsing with dichloromethane, the initiator-grafted silicon chip was stirred in a solution of CuBr/CuBr_2_/bipyridine/sodium methacrylate at 40 °C under N_2_ protection for 2–4 h. The polymer brushes of sodium polymethacrylate^47^,^48^,^49^,^50^,^51^ were grown only within the nanopits, with a dry thickness of 10–20 nm above the silicon surface (cartoon in Fig. 1d and AFM image in Fig. 1D). The use of the sodium methacrylate salt monomer and ATRP provides advantages in that the brushes can grow more densely and homogeneously to a controllable height by regulating the monomer and catalyst concentrations^52^,^53^, the polymerization is performed in aqueous media, and finally, acidification can convert all sodium salts into acids for further applications. SI-ATRP was performed in a water/methanol (50:50 v/v) solvent in which methanol was used to improve the wettability of the hydrophobic nanopits for direct contact with monomers to grow polymer brushes. Similar to the literature^53^, the polymer brushes grew slowly after 2 h under our reaction conditions; therefore, a longer polymerization time, up to 4 h, did not substantially change the dot morphology but could increase the plumpness of the dots.

**Figure 2:**
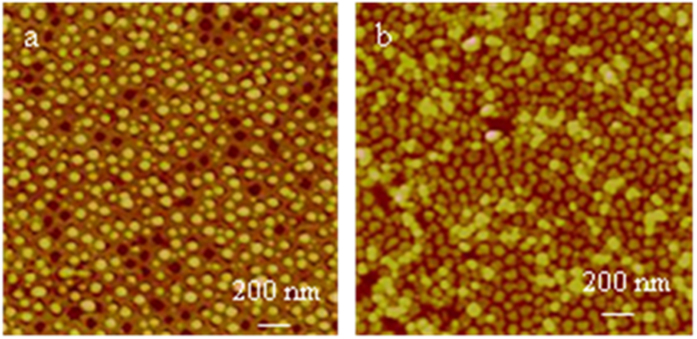
“Defects” of the PMAA nanodot array: (**a**) PMAA nanodot array with some empty nanopits, and (**b**) two layers of PMAA nanodots.

Statistical analyses were performed on Fig. 1A–D (for Gaussian analyses, see Figs S6-S8 in the SI). The dimensions of the P4VP micellar core and PS corona can be tuned by adjusting the molecular weight of PS-*b*-P4VP. For the PS-*b*-P4VP with a MW of 109,000-27,000 g/mol in Fig. 1A, the center-to-center spacings of the hexagonally packed dots range from 90 to 130 nm, with an average of 110 nm. The P4VP dot diameters range from 9 to 75 nm, with an average of 42 nm, and the heights range from 15 to 35 nm, with an average of 25 nm. Analysis of Fig. 2B reveals the center-to-center spacings of the SiH*x* pits between 90 and 120 nm (centered at 115 nm), diameters between 16 and 36 nm (centered at 26 nm), which is half that of the P4VP dots in Fig. 1A, and depths between 10 and 16 nm (centered at 13 nm). Thus, the orthogonal double arrays with SiH*x*-covered pits and the top flat native SiO_2_ layers were available for surface-selective hydrosilylation reactions. After the initiator monolayer of α-bromoisobutyrate was grafted, no significant topomorphological difference was found in Fig. 1C compared to Fig. 1B because the theoretical thickness of the initiator monolayer was 1.2 nm. Finally, analyses of the PMAA brush dots in Fig. 1D show the center-to-center spacings between 90 and 130 nm (centered at 110 nm), the diameters between 20 and 80 nm (centered at 50 nm), and the heights between 6 and 20 nm (centered at 13 nm). The dots of Fig. 1D are nearly 2 times larger than the pits in Fig. 1B,C due to expansion of the brushes. The brush domains are circular-like dots with irregular stretching tips due to diffusion-limited growth, and these dots are not exactly the same as the perfectly round P4VP dots with sharp boundaries in Fig. 1A.

### “Defects” in the PMAA nanodot array

**Figure 3:**
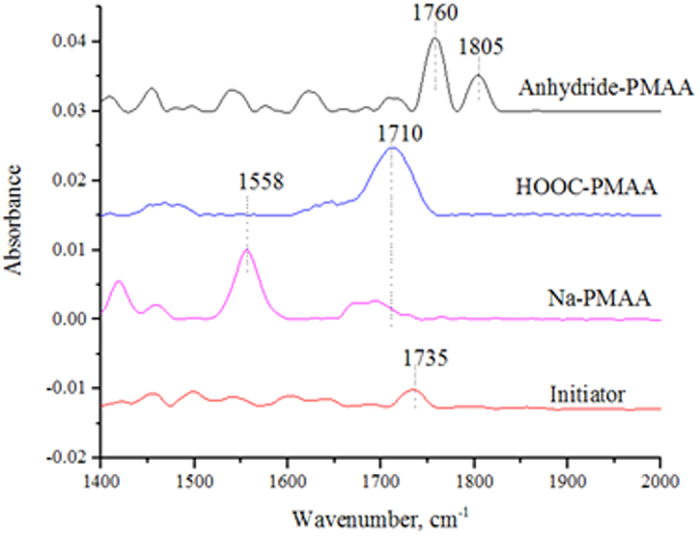
MTR-IR spectra. α-bromoisobutyrate-ended initiator monolayer (Initiator), sodium polymethacrylate (Na-PMAA), PMAA (HOOC-PMAA), and polymethacrylic anhydride (Anhydride-PMAA).

Actually, every fabrication step has a probability for success and failure, even under the optimal reaction conditions. Except for the perfect nanodots in Fig. 1D, we observed variable topomorphologies of PMAA nanodots grown from the nanopit templates, which were defined as non-polymerization (empty nanopits with nearly no polymers, Fig. S4a in the SI), off-polymerization (partly-filled nanopits with polymers, Fig. S4b in the SI), proper-polymerization (fully-filled nanopits with polymers as nanodots in regular shapes, Fig. S4c in the SI), over-polymerization (over-filled nanopits with polymers but in irregular nanodot shapes including two-layer nanodots, Fig. S4d in the SI), and mixed nanodots and nanopits (both nanodots and nanopits coexisted on the same chip, Fig. S4e in the SI). To discuss the uncertainties and yields during the fabrication procedure, we defined a successful chip as “with over 80% nanopits converting to regular nanodots protruding out of the silicon surface horizon”. As shown in Fig. 1a to d, the stepwise procedure for the chip fabrication includes three steps: wet etching to fabricate regular nanopits covered with SiH*x* species, microwave irradiation to execute the surface hydrosilylation reaction and to generate molecular monolayers with end-functionalized initiators within the nanopits, and ATRP in solution to grow polymer brush nanodots from the nanopit templates. At the beginning stage of this research project, we characterized 20 chips (20 × 20 mm^2^) after each fabrication step. The topomorphologies of the chip after the first and the third steps can be well distinguished by AFM imaging. The first wet etching step had a yield of 90% (18 chips had regular nanopits, which were independent and isolated, and with round shapes distributed between 16 and 50 nm in diameter; whereas only 2 chips had either shallow pits by underetching or irregular and connected nanopits by overetching), evaluated from AFM imaging. While after the second step, the nanopits grafted with 1.2 nm thick molecular monolayers cannot be distinguished by AFM from their precursor nanopits, therefore we applied MTR-IR to evaluate the quality of initiator monolayers within the nanopits. A standard was set that the carbonyl stretching band at 1735 cm^−1^ should be detected (see the bottom trace in Fig. 3); at such a standard level, 13 chips were detected to have the carbonyl signal (a yield of 72% ). The detectable initiator chips with MTR-IR indicated that the surface coverage of grafted initiators was qualified for further proper ATRP to grow polymer brushes. Further ATRP polymerization of the 13 chips obtained 9 chips with plump PMAA nanodots (a yield of 69%, including 6 proper-polymerization chips and 3 over-polymerization and mixed nanodots and nanopits chips), and 4 chips with off-polymerization nanodots in the transition states, screened with AFM. The rest of 5 non-qualified initiator chips, having less surface coverage of initiators, still induced ATRP within the nanopits but with a lower polymer growth rate; as a result, these chips were finished at the off-polymerization or non-polymerization states. While to check every chip at each step with AFM and MTR-IR was troublesome, after determining the optimal reaction conditions, we ran all three step reactions on a chip (10 × 10 mm^2^) continuously, without monitoring the first wet etching with AFM and the second initiator grafting with MTR-IR, and only finally scanned the chip with AFM after the third ATRP step. We did statistic analysis on 100 samples (chips): 30% proper-polymerization chips with regular nanodots, 15% non-polymerization ones with empty nanopits, 40% off-polymerization ones in transition states, 7% over-polymerization ones with irregular nanodot shapes including two-layer nanodots, and 8% mixed nanodots and nanopits ones.

Except for some successful chips with perfect nanodots via proper-polymerization, other chips had nanostructural “defects”. Two most typical defect morphologies are shown in Fig. 2. Figure 2a shows approximately 85% dots with polymer brushes and 15% empty pits without polymer brushes. These empty nanopit “defects”, along with the tentative observation of many dots bearing interstitial interfaces with their corresponding pits, rather than the perfect dots in Fig. 1D, clearly demonstrate that the PMAA brushes grew from only the nanopits. The dot diameters are distributed between 40 and 80 nm (centered at 60 nm), the center-to-center spacings are between 90 and 150 nm (centered at 120 nm), and the dot heights are between 10 and 30 nm (centered at 20 nm). The discrepancy in dot size parameters, such as diameter and height, between Figs 1D and 2a is due to the experimental variations between batches. The following are possible reasons for generating the empty pits: 1) the dots were washed away during the ultrasonication cleaning procedure due to the breaking of the binding between polymer brushes and the intaglio surface, thus lifting off the dots from their sitting pits, which are the most possible reason; and 2) the lower SiH*x* density and the followed lower initiator density in the pits, or air bubbles covering the pits, could not initiate the growth of PMAA brushes, which are the least possible reasons.

**Figure 4:**
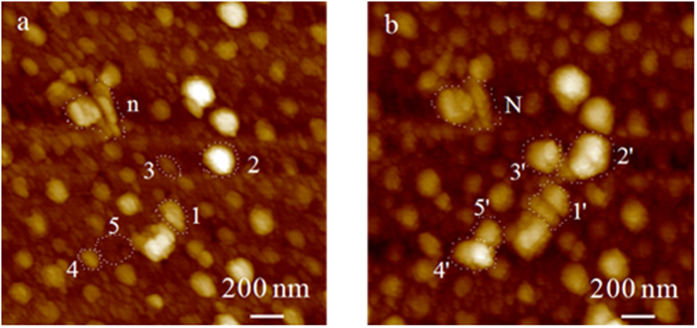
AFM images of pH stimulus of PMAA nanodots. Two corresponding AFM images of PMAA dots at pH 3.0 (**a**) and 9.0 (**b**) were scanned in the same region, illustrating the PMAA dot swelling and topomorphological evolution. The swelling volume factor estimated from unlabeled dots is 2.0, whereas that for the Arabic number labeled pairs of 1′/1 and 2′/2 is 5.0, for 3′/3 and 4′/4 is 10, and for 5′/5 is infinite. For the interpretation, please see the text.

Another specific type of “defects” is shown in Fig. 2b. A significant feature is the two layers of PMAA dots. The lower-layer dots show polygon shapes, which are slightly different from the circular-like shapes in Figs 1D and 2a, and a nanopore of approximately 5 nm in diameter at the center of nearly every dot. The dot diameters range from 30 to 90 nm (centered at 60 nm), the center-to-center spacings range from 70 to 110 nm (centered at 90 nm), and the heights range from 0 to 10 nm (centered at 5.0 nm) (Fig. S10 in the SI). Due to batch-to-batch variations, the lower-layer dots have a higher density than in Figs 1D and 2a; thus, they appear to be more crowded in the same scale size. The upper-layer dots have an average diameter of approximately 70 nm and are approximately 13.0 nm high. Figure 2b illustrates that PMAA brushes grew primarily around the edges of the pits, leaving a nanopore at the dot center. The edge-prone growth should be attributed to the higher density of surface SiH*x* species, therefore having a high density of initiators at the edges. The two-layer dot phenomenon can be interpreted as follows: the polymer chains at the pit edges grew horizontally towards each other initially, and the interchain repulsive interactions forced them to extend up somehow and to maximize the distance between each other (that is why the lower layer is above the silicon surface horizon), however the faster circumferential convergence growth reached much denser polymer frontiers due to the limited space in the center before they fused together, the highly dense frontiers rendered ATRP much more slowly towards each other in the horizontal direction, once new polymer tips sprout out of some sites of the lower-layer nanodots, they grew much faster in the vertical direction upwards due to the diffusion-limited aggregation; whereas the frontiers of the lower-layer dots stopped growing or had a very slow growth rate, which explains why a 5 nm nanopore sat at the center of every lower-layer dot. The diffusion-limited aggregation, well known in physics, means that monomers diffuse into the depletion zone and then deposit and grow at the brush tips much faster than in any other location^54^,^55^. The diffusion-limited growth mechanism in polymer brushes, theoretically predictable^56^ but lacking evidence, remains less explored. Our approach using separated polymer brush dots at the nanometer scale but in the mass and very-large-scale production renders the tentative observation of two-layer polymer brush structures realistic. Because the ATRP of sodium methacrylate occurred linearly on all polymer chains, the upper-layer dots grew via diffusive growth without branching, resembling vertical cylinders, unlike the most common dendritic structures^54^,^55^,^56^. Currently, we cannot control their growth, but we suggest that the growth of two-layer brushes is related to the reactivity and concentration of catalysts, monomer concentrations, and polymerization speeds. Even for the one-layer dots, when they were imaged at pH 9.0 by liquid AFM (Fig. 4), the sub-nanodomains can be well distinguished in one nanodot, which can also be classified into the nanostructures regulated by diffusion-limited growth. Such delicate nanostructures illustrate the necessity to investigate the growth mechanisms of polymer brushes for the better control of uniform films.

Other polymer brush dots, such as poly (2-hydroxyethyl methacrylate) (PHEMA) and poly (N-isopropylacrylamide) (PNIPAM), were also grown on the nanopit templates, as shown in Figs S11 and S12 of the SI, respectively. Due to the different molecular behaviors, part of the PHEMA brush dots is easily smeared on the surface; PNIPAM brush dots appear higher and denser but with irregular shapes.

### MTR-IR characterization

As AFM cannot distinguish the covalently grafted initiator monolayer nanopits from the HF wet-etched ones, an additional characterization method is needed. Fortunately, a semiconductor surface-sensitive technology, MTR-IR, developed in our laboratory, is powerful for measuring the molecular monolayers or submonolayers on semiconductor surfaces. The signal of SiH*x* etched from the planar silicon crystalline (100) face, composed of three stretchings, SiH, SiH_2_, and SiH_3_^44^, cannot be detected because the bands appearing as a bump are not sufficiently strong to be distinguished from noise. The SiH*x* signal etched from a planar Si(111) can be easily detected with MTR-IR due to a sharp SiH band at 2083 cm^−1^ 39. Because Si(100) is predominantly used in solar cell and integrated circuit productions, we used Si(100) rather than Si(111) as substrates in our study. MTR-IR was used to analyze the weak signal of initiator monolayers grafted on the nanopits (for the 20 × 20 mm^2^ chip, the infrared beam simply transmits the chip 4 times)^38^,^39^,^40^. MTR-IR can detect the weak absorbance signal of the carbonyl stretching at 1735 cm^−1^ (the bottom trace in Fig. 3) and the stretchings of CH_2_ at 2852 and 2922 cm^−1^ (Fig. S1 in the SI). The maximum signal of the antisymmetric CH_2_ stretching at 2922 cm^−1^ measured was not over 0.008, indicating a monolayer or submonolayer of the initiator sitting within the nanopits. Fig. 3 shows the infrared spectra of initiator (Initiator), sodium polymethacrylate (Na-PMAA), polymethacrylic acid (HOOC-PMAA), and polymethacrylic anhydride (anhydride-PMAA) in the carbonyl stretching region of the most interest. The polymer brushes of sodium polymethacrylate provide the carboxylate stretching band at 1558 cm^−1^. After the sodium polymethacrylate chip was soaked in a pH 3.0 solution for 1 h, the carboxylic acid peak appeared at 1710 cm^−1^. PMAA brushes are one of the most common matrices applied to immobilize proteins and other biomolecules. However, as we reported, its activation intermediate of N-ethyl-(3-(dimethylamino)-propyl)carbodiimidehydrochloride/N-hydroxysuccimide (EDC/NHS) is an anhydride, which results in a lower amidation yield (the amidation efficiency can be improved by reiterating the EDC/NHS activation and amidation several times)^57^,^58^. The anhydride peaks at 1760 and 1805 cm^−1^ after activation by EDC/NHS are shown in Fig. 3 for further demonstration of the PMAA brush dots. Additional MTR-IR spectra of PNIPAM and PHEMA brushes are recorded in Fig. S2.

### Stimulus response of PMAA nanodots to pH

One of the most exciting features of polymer brushes is their stimulus response. The switching of the adaptive and responsive brush interfaces can be triggered by an external stimulus, such as changes in pH, temperature, mechanical force, or light^17^. PMAA is a highly pH-responsive material. When the pH is at 3.0, PMAA contracts, and its chain collapses. When the pH is at 9.0, the COOH becomes carboxylate; thus, PMAA chains are negatively charged and elongate due to Coulomb repulsion. The reversible switching can tune the physical properties of the reconstructable surfaces, such as their wettability, permeability, adhesion, adsorption, mechanical and optical properties, for versatile applications.

Fig. 4 presents two PMAA dot images in the same region at pH 3.0 (a) and 9.0 (b), which were obtained by fixing the AFM tip at the same position of a chip in a drop of 90 μL solution at pH 3.0 and 9.0 titrated using HCl or NaOH solutions. PMAA dots in Fig. 4a were first imaged at pH 3.0; then, a small volume of concentrated NaOH was injected with a microsyringe, and the solution remained for 30 min for electrolyte diffusion and PMAA swelling equilibrium; finally, the swollen PMAA dots in Fig. 4b were imaged. The PMAA dots at pH 9.0 with normal swelling are approximately 20% larger in diameter and 10% larger in height, corresponding to previous reports^37^,^47^,^48^,^49^,^50^,^51^,^59^,^60^,^61^. We labeled five pairs of dots with Arabic numbers (1–5 in Fig. 4a corresponding to 1′-5′ in Fig. 4b, respectively), which showed considerably more swelling than the unlabeled normal dots. The swelling phenomena of polymer electrolytes, especially PMAA, have been extensively investigated. Most reports gave a linear swelling factor (length at pH 9.0/length at pH 3.0) of 1.4-6 because the brushes were grown on a planar chip at the macroscale^37^,^47^,^48^,^49^,^50^,^51^,^59^,^60^,^61^. Instead, in this study, we used a spherical model rather than a linear one to calculate the volume swelling factor because these brush nanodots can laterally extend in a more flexible manner than they can at the macroscale. The lateral diameter of a dot can be considered as that of a dot sphere. The volume swelling factor of pH 9.0/pH 3.0 for regular dots, averaged over 25 nanodots, is 2.0, which is in accordance with the previous reports using ellipsometry, quartz balance, and other technologies^47^,^48^,^49^,^50^,^51^. The volume swelling factor for two pairs of 1′/1 and 2′/2 is 5.0, for 3′/3 and 4′/4 is 10.0, and for 5′/5 is infinite. The larger volume expansion effect can be attributed to two causes: a buried dense polymer portion in deeper pits for 1-1′ and 2-2′, and random docking of other free-floating dots for 3-3′, 4-4′, and 5-5′. The free-floating nanodots might originate from other regions of the chip during the pH switching process. The volume, the dot density and the topomorphologies can be well distinguished from pH 3.0 to pH 9.0. At pH 3.0, the dots are considerably denser, and their outermost topologies appear solid and robust. At pH 9.0, every dot appears to have a faint halo around it, probably due to the extended brush tips associated with more water. The subdomains and their boundaries can be well distinguished, e.g., in the corresponding dots of N from n, 1′ from 1, and 2′ from 2. The extended PMAA chains at pH 9.0 allow the AFM tips to draw a considerably better geomorphologic map of the brush dot In Fig. 4b. Because no missing dots were identified in Fig. 4b from Fig. 4a, we are confident that the one-end fixed dots were robust during AFM scanning, pH changes, and other manipulations. In sum, the stimulus-responsive switching can be well imaged and distinguished by AFM under the two static conditions of pH 3.0 and 9.0.

### Growth of PMAA nanorods

**Figure 5:**
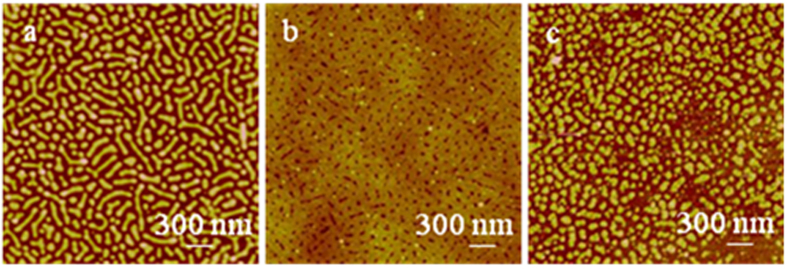
Rod-like arrays from a high molecular weight of PS-*b*-P4VP (330,000-125,000 g/mol): (**a**) P4VP rods surrounded by PS corona, (**b**) wet-etched nanoslits after relieving PS-*b*-P4VP, and (**c**) PMAA brush rods grown via SI-ATRP.

The typical patterning structure for PS-*b*-P4VP is the hexagonal close-packed dot array of micelles. However, by increasing the molecular weights of both PS and P4VP over 100,000 daltons (for example PS: 330,000, P4VP: 125,000), the P4VP aggregates tend to form the rod array in Fig. 5a. It is difficult to etch the same nanoslit patterns, mirroring the nanorods of Fig. 5a, due to the inhomogeneity of the nanorod structures to some degree. The rods are composed of loose connections of dense P4VP dots, which can be observed from the pairs of nanopits in the nanoslits of Fig. 5b. In this case, wet etching can easily go to two extremes: overetching produces porous silicon, whereas underetching generates nanopits. Only an appropriate etching can form the rod-mirrored slit patterns, as shown in Fig. 5b. Following the same protocol for the growth of polymer brushes, rod-like patterns were successfully produced in Fig. 5c.

Statistical analyses (Fig. S13 in the SI) show that in Fig. 5a, the rod width is approximately 50 nm, the lengths range from 50 to 600 nm, and the height is approximately 20 nm. After etching, round pits and pitted slits can be observed in Fig. 5b with comparable ratios (Fig. S14 in the SI). Following the growth of polymer brushes, PMAA rods and dots, which were comparable in counts, were formed in Fig. 5c. They have an average width of 60 nm, lengths from 60 to 350 nm, and an average height of 17 nm (Fig. S15 in the SI). The PMAA rods are shorter in length than P4VP nanorods in Fig. 5a because they followed the nanoslit patterns in Fig. 5b, whereas they are plumper than the nanoslits due to expansion of PMAA brushes.

### Growth of embossed nanofingerprints of PMAA

For the application of nanotechnology, many types of nanodevices are rudimentary. In addition to the nanodot array, other patterns, such as nanowire connections or devices, are also in demand. As reported, the fingerprinting patterns can be generated on only the PS-*b*-P2VP-coated silicon surface by solvent annealing, whereas they cannot be generated from PS-*b*-P4VP^9^,^10^. A possible interpretation of this result is that PS-*b*-P4VP cylinders contact the silicon surface directly and possess strong interactions and high adhesion forces, whereas P2VP cylinders do not. Thus, the P2VP dotted aggregates can migrate to form the fingerprinting patterns during solvent annealing. For example, the PS-*b*-P2VP dot array can be easily converted to a regular fingerprints array under the saturated THF/H_2_O vapor. Our efforts with wet etching on the PS-*b*-P2VP template to generate nanofingerprints were unsuccessful. This approach often generated irregular porous silicon structures, likely due to two factors: 1) the P2VP nanowires were not fully connected continuously without any breakings but were composed of loosely connected P2VP nanodots; and 2) an underlying ultrathin PS layer was in close contact with silica; therefore, aqueous HF solutions diffused everywhere equally and etched silicon into porous structures with different dynamics.

An alternative approach to generate nanofingerprints on silicon is to etch the dotted platinum particulate nanowires with the metal-assisted stain etch^10^,^62^,^63^,^64^,^65^. Using an etching solution of HF/H_2_O_2_/EtOH (1:1:3 v/v/v) for 3–6 min, the platinum nano-particles were etched away, and shallow parallel nano-ditches were obtained. The etching solution concentration and etching time were crucial to produce the nanofingerprints. Overetching generated porous structures, whereas underetching did not form the ditches and did not etch away the platinum particles. Even an appropriate etching cannot remove all platinum particulate residues. After surface hydrosilylation chemistry to graft the initiators, ATRP with methacrylate sodium generated embossed nanofingerprints.

**Figure 6:**
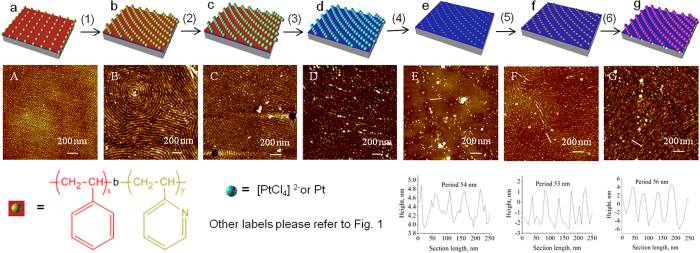
Schematic procedure for the fabrication of embossed nanofingerprints of PMAA and corresponding AFM images. Their corresponding cartoons and AFM images are shown as: (**a** and **A**), micellar diblock copolymer nanodot array of PS-*b*-P2VP; (**b** and **B**), solvent-annealed PS-*b*-P2VP fingerprints in a THF/H_2_O = 10:1 solution for 36 h; (**c** and **C**), platinichloride-deposited fingerprints after immersion in a 0.9% HCl and 70 mmol/L Na_2_PtCl_4_ solution for 3 h; (**d** and **D**), platinum dotted fingerprints after O_2_ plasma for 60 s at 30 W to remove PS-*b*-P2VP; (**e** and **E**), SiH*x* pendant fingerprints after etching in a HF/H_2_O_2_/EtOH (v/v/v = 1:1:3) solution for 3–6 min; (**f** and **F**) the initiator fingerprints; (**g** and **G**), PMAA brush fingerprints. The six steps are as follows: (1) solvent annealing, (2) platinichloride incubation, (3) O_2_ plasma etching, (4) wet etching, (5) initiator grafting, and (6) SI-ATRP. The physical meanings of the schematic labels different from Fig. 1 are illustrated at the bottom.

Figure 6 depicts the schematic fabrication procedure with cartoons and their corresponding AFM images in each step respectively. A micellar toluene solution of PS-*b*-P2VP (0.5% w/w) was spin-coated onto silicon, forming a dot array of P2VP (cartoon in Fig. 6a and AFM image in Fig. 6A). Statistical analyses (Fig. S16 in the SI) show that their dot diameters are distributed between 5 and 25 nm (centered at 15 nm), the center-to-center spacings range between 15 and 45 nm (centered at 30 nm), and the dot heights range between 1.4 and 4.0 nm (centered at 2.7 nm). After solvent annealing by THF/H_2_O (10:1 v/v) for 30–40 h, the PS-*b*-P2VP dot array was converted into fingerprinting patterns (cartoon in Fig. 6b and AFM image in Fig. 6B, cross section profile in Fig. S17 of the SI) in which the bright line width is approximately 20 nm, the distance between neighboring lines is 59 nm, and the height of the bright line is 5 nm. After immersion in a platinum salt solution (0.9% HCl, 70 mmol/L Na_2_PtCl_4_) for 3 h, the protonated cationic P2VP dots and anionic PtCl_4_^2−^ were attracted to each other due to electrostatic forces. Therefore, deposition of PtCl_4_^2−^ caused the bright P2VP nanowires to swell to a width of approximately 40 nm (cartoon in Fig. 6c and AFM image in Fig. 6C, cross section profile in Fig. S18 of the SI). The average height of these stripes is ~4 nm, similar to that in Fig. 6B. Special attention should focus on the indented shallow pits in the bright-widened P2VP fingerprints, indicating the deposition of platinichloride. The platinichloride-deposited silicon chip was exposed to O_2_ plasma, and then, all polymer components of PS-*b*-P2VP were removed, whereas platinates were reduced to platinum particulate nanowires (cartoon in Fig. 6d, AFM image in Fig. 6D, and cross section profile in Fig. S19 of the SI). The platinum nanoparticles were aligned along the P2VP dotted nanowire template, and each platinum nanoparticle corresponds to one of the P2VP nanodots. The distance between neighboring nanowires is approximately 58 nm. The diameter of the platinum dots is 7.0 nm. The size of the platinum nanoparticles was primarily related to the immersion time, where a longer time resulted in larger particles. However, the maximum size was limited by the diameter of P2VP cylinders of 15 nm. Metal-assisted stain etching with an etchant of HF/H_2_O_2_/EtOH (1:1:3 v/v/v) at 40 °C for 3–6 min provided the SiH*x* pendant nanofingerprints (cartoon in Fig. 6e, AFM image in Fig. 6E, the cross section profile of the marked line in Fig. 6E inserted under Fig. 6E, more AFM images in Fig. S20 of the SI). The etching, resembling a galvanic cell by injecting holes into the valence band of Si from the platinum particles, facilitated the local oxidation and dissolution of Si atoms around the platinum dots. The platinum nanoparticles were mostly consumed, whereas some agglomerated and grew into large particles during the etching period, explaining the observation of some large particles in Fig. 6E. These particulate debris cannot be easily removed. In Fig. 6E, the fingerprinting patterns have considerably less contrast in height, only 1 nm difference from the bright to the dim lines, the bright lines have an average width of 5–7 nm, and the periodic distance between neighboring lines stays the same (54 nm). Considering the 7.0 nm diameter of the platinum dots, we assigned the bright lines to the underlying nanostructures of the platinum dots covered by SiH*x* species. We speculate that the dim line array with fewer SiH*x* species is due to faster etching in the nano-valley between the particulate platinum wires, whereas the bright line array is due to slower etching of the underlying platinum nanodots, generating very fine nanoporous lines with richer SiH*x* species. As we reported, Pt-assisted chemical etching provides higher hydrosilylation efficiency than does dilute HF etching because of the presence of more SiH_2_ species^44^. After the initiator of 10-undecen-1-yl-2-bromo-2-methylpropionate was grafted under microwave irradiation, the fingerprints in Fig. 6F (cartoon in Fig. 6f, AFM image in Fig. 6F, the cross section profile of the marked line in Fig. 6F inserted under Fig. 6F, more AFM images in Fig. S21 of the SI) became more distinguishable with a periodic distance of 53 nm, and the chip surface was cleaner due to the removal of the platinum particulate debris. The bright lines are 2-3 nm higher than the dim lines, indicating that the initiator of α-bromoisobutyrate (1.2 nm high) was grafted onto the bright line of Fig. 6E. After SI-ATRP of sodium methacrylate, the PMAA nanofingerprints were fabricated, as shown in the cartoon of Fig. 6g and in the AFM image of Fig. 6G (the cross section profile of the marked line in Fig. 6G inserted under Fig. 6G and more AFM images in Fig. S22 of the SI). The periodic distance between neighboring lines is 56 nm, the average bright line width is approximately 30 nm, and the average line height is 8.0 nm. The MTR-IR spectra of the initiator in Fig. 6F and of PMAA sodium salts and acids in Fig. 6G were recorded in Fig. S3. The fingerprint fabrication procedure is delicate. Its success mainly depends on the critical wet-etching of Pt-particulate lines with the HF/H_2_O_2_/EtOH solution to generate the low-contrast fingerprints of Fig. 6E.

## Conclusion

We successfully grew embossed nanodots, nanorods, and nanofingerprints of PMAA brushes on silicon from their corresponding templated nanostructures with pendent SiH*x* species, which were wet-etched from block copolymers. The critical steps were wet-etching and surface hydrosilylation chemistry in which the former generated SiH*x* pendant nanostructures and the latter produced the initiator monolayer with a high surface coverage on the SiH*x* pendant nanostructures. MTR-IR was applied to characterize the initiator monolayer of α-bromoisobutyrate. The densely grafted initiator monolayer with a clear carbonyl stretching band can efficiently initiate SI-ATRP to form successful embossed nanostructures. Due to the unique feature of block copolymer lithography of mass and very-large scale production, we observed the two-layer nanodot structures of PMAA. Such a two-layer nanodot phenomenon experimentally demonstrates the growth difference of polymers within nanopits from on a planar substrate and on a micro-/nano-sphere. Polymers grow on a planar substrate and on a micro-/nano-sphere always in the same direction and have large space for extension, whereas the polymers grow at the nanopit towards each other initially, then reached much denser frontiers in the center before they fuse together, the highly dense frontiers rendered ATRP to stop or to grow much more slowly towards each other, once new polymer tips sprout out of some sites of the lower polymer layer, they will grow much faster in the perpendicular direction with regard to the lower layer growth, due to the diffusion-limited aggregation, and thus form the second polymer nanodomain layers. The pH-stimulated switching of PMAA chains between expanding and contracting was also observed by liquid-phase AFM. Its volume at pH 9.0 will generally swell to twice that at pH 3.0. However, a large volume expansion of PMAA with volume swelling factors of 5, 10, and infinite were observed for some dots, which were attributed to the buried dense polymer portion in deeper nanopits and random docking of free-floating dots. More importantly, the subdomains and their boundaries in a nanodot on the nanometer scale can be distinguished very well at pH 9.0 by liquid AFM, which were not visible in air and were ambiguous in the liquid phase at pH 3.0. In addition, embossed PMAA nanorods were achieved from a high-molecular-weight PS-*b*-P4VP template. Finally, we succeeded in the fabrication of nanofingerprints of PMAA on wet-etched silicon from platinum particulate fingerprints via modification of the PS-*b*-P2VP lithography.

The surface-selective functionalization and nanopatterning of silicon by polymer brushes via block copolymer nanolithography in large-scale and mass production combine the potent functionalities of integrated silicon devices and biologically compatible materials responding to environmental cues together. These materials have many potential applications. For example, the nanodots can be applied to biochips, where a straightforward application is next-generation sequencing. In such a sequencing chip, every spot corresponds to a DNA sequence of several tens to hundreds of base pairs^66^,^67^,^68^. After measuring enormous sequences on a chip, the sequence assembly using algorithms and software will result in the whole-genome sequencing. The spot density per area of our PMAA nanodot chip will achieve up to at least 10^8^ spots/mm^2^, whereas that of the current Illumina sequencing technologies is 7 × 10^5^ spots/mm^2^. Therefore, the sequencing chip based on our technology will contain 100 times more sequencing data per area than the current technologies, whereas retaining more DNA molecules and higher imaging efficiencies per spot due to its 3-dimensional volume. To act as both buffer layer and multiplex electrodes, the polymer brush nanopatterns combined with silicon integrated circuits should be ideal for brain chips, neurochips, tissue chips, and organ chips in the human body and in other living organisms^4^. In the semiconductor industry, the integrated hybrid devices could be used in silicon photonics, optoelectronic devices such as solar cells, bit patterned media, polymer electronic memories, and organic-lighting diodes. Because it is an application-oriented technology, the integration of the growth of polymer brushes with block copolymer nanolithography will have a prosperous future.

## Additional Information

**How to cite this article**: Lu, X. *et al.* Growing Embossed Nanostructures of Polymer Brushes on Wet-Etched Silicon Templated via Block Copolymers. *Sci. Rep.* **6**, 20291; doi: 10.1038/srep20291 (2016).

## Supplementary Material

Supplementary Information
