# Supplementary material for: Growing Embossed Nanostructures of Polymer Brushes on Wet-Etched Silicon Templated via Block Copolymers
Source: Sci Rep. 2016 Feb 4;6:20291. doi: 10.1038/srep20291 (PMC4740862; doi:10.1038/srep20291)
Supplement: Supplementary Information [file srep20291-s1.pdf]

Supplementary Information

Growing Embossed Nanostructures of Polymer Brushes on Wet-Etched Silicon Templated via Block Copolymer

Xiaobin Lu, Qin Yan, Yinzhou Ma, Xin Guo, Shou-Jun Xiao*

State Key Laboratory of Coordination Chemistry, Collaborative Innovation Center of Chemistry for Life Sciences, School of Chemistry and Chemical Engineering, Nanjing University, Nanjing 210093, Jiangsu, China

*Corresponding author: sjxiao@nju.edu.cn, Tel.: +86-025-83621001 and fax: +86-025-83314502.

1. Experimental

Materials. Single-side and double-side polished silicon wafers ((100), p-type, boron-doped, 5.0-8.0 Ω.cm, 500 μm thick) were purchased from Shanghai Junhe Electronic Materials Technology Co. Ltd. Sodium methacrylate, methyl methacrylate, 2-hydroxyethyl methacrylate, bipyridine, ω-undecylenyl alcohol (98%), 2-bromoisobutyryl bromide (98%), $Na_2PtCl_4.xH_2O$ were from Alfa Aesar. N-Isopropylacrylamide (NIPAM, 99%), pentamethyldiethylenetriamine (PMDETA, 98%), copper (II) bromide ($CuBr_2$, 98%), and copper (I) bromide (CuBr, 98%) were from Aldrich. Water (18 MΩ.cm) was from a Milli-Q Ultrapure Water Purification System. Polystyrene-*block*-poly(4-vinylpyridine) (abbreviated as PS-*b*-P4VP) with MWn of 109000-27000 (P3910-S4VP) and 330000-125000 (P5733-S4VP) and polystyrene-*block*-poly(2-vinylpyridine) (abbreviated as PS-*b*-P2VP) with MWn of 56000-21000 (P4096-S2VP) were from Polymer Source Inc. of Canada.[1-3]

Pretreatment of Wafer. The wafer was cut into 10×10 mm^2 and 20×20 mm^2 pieces. Silicon chips were immersed in a piranha solution (3:1 H_2SO_4/H_2O_2) at the mild boiling temperature for 3 h to remove any organic residues. The cleaned chips were rinsed extensively with water and ethanol and subsequently dried in a stream of nitrogen. The chips were then cleaned via the standard RCA cleaning procedure sequentially: immersed in a hot solution of 5:1:1 (v/v/v) $H_2O/NH_4OH/H_2O_2$ for 15 min, rinsed with excess water, immersed in a hot solution of 6:1:1 (v/v/v) $H_2O/HCl/H_2O_2$ for 15 min, rinsed with excess water, finally dried with a stream of

nitrogen before polymer spin-coating.[2,4,5]

Preparation of Block Copolymer Micellar Solution. PS-*b*-P4VP (109000-27000) was stirred at 80 ℃ in toluene overnight, a selective solvent for the PS block, to make a 0.5% (w/w) micellar solution, and then allowed to cool to room temperature.[5-7] With the same protocol, PS-*b*-P4VP with MWn of 330000-125000 was prepared to a 0.25% (w/w) micellar solution. PS-*b*-P2VP with MWn of 56000-21000 was prepared to 0.5% (w/w) micellar solution in toluene but stirred at room temperature overnight. The micellar solution was allowed to equilibrate for at least 1 day prior to use.[1-4,8]

Nanopits or Nanorods Preparation Templated from PS-b-P4VP Films. For PS-*b*-P4VP, 15 μL of the polymer solution was dropped on a 10×10 mm^2 chip and spin-coated at 3300 rpm for 60 s (or 60 μL micellar solution dropped on a double-side polished 20×20 mm^2 chip and spin-coated at 3300 rpm for 60 s) in an ambient environment (KW-4A model Spin Coater, Institute of Microelectronics of Chinese Academy of Sciences). The nanopits were etched from the PS-*b*-P4VP (109000-27000)-coated chip in a 0.4% HF (aq) for 5 min or 0.1% HF for 30 min at room temperature, while nanorods (PS-*b*-P4VP, 330000-125000) in a 0.4% HF for 8 min at 40 ℃.[5-7] After etching, the chip was thoroughly rinsed with water and dried under a nitrogen stream. Then, the PS-*b*-P4VP film was removed in toluene and trichloromethane sequentially by 15 min ultrasound bathing respectively. Finally the clean chips with the SiH_x pendant nanopits array were dried under a nitrogen stream.[1-3,5]

Fingerprints Etching Templated from PS-b-P2VP Films. The PS-*b*-P2VP micellar solution (10 μL) was dropped on a 10×10 mm^2 chip and spin-coated at 3500 rpm for 60 s in an ambient environment. Subsequent solvent-annealing to form fingerprints was carried out at room temperature in the presence of vapor from an 11 mL of 10:1 THF/H_2O mixture for 30-40 h in a 1.9 L desiccator. Since our effort to wet-etch PS-*b*-P2VP fingerprints did not gain the fingerprinting patterns, we detoured to succeed by etching the Pt-dotted fingerprints. To obtain Pt-dotted fingerprints, the PS-*b*-P2VP fingerprints chip was immersed in a 70 mmol.L^{-1} Na_2PtCl_4 and 0.9% HCl (aq.) solution, held in a Teflon beaker for 3 h, then rinsed with excess water, dried under a nitrogen stream, and dry-etched by oxygen plasma to remove PS-*b*-P2VP and reduce palatinates to platinum nanodots at 50 mtorr, 50 s.c.c.m., and 30 W for 60 s (Oxford Instruments Plasmalab 80 Plus).[1-4,6,8] Finally the Pt-dotted fingerprinting chip

was immersed in a 1:1:3 (v/v/v) HF(40%)/H_2O_2/EtOH for 3-6 min at 40 ℃, thoroughly rinsed with water, and dried under a nitrogen stream.

Initiator Synthesis.[9-12] The initiator of 10-undecen-1-yl 2-bromo-2-methylpropionate was synthesized from 10-undecen-1-ol (8.3 mL, 41 mmol in 41 mL dry THF) by addition of 2-bromoisobutyryl bromide (5.1 mL, 41 mmol) and pyridine (3.5 mL, 43 mmol). The reaction mixture was stirred for 15 h under N_2, then n-hexane (25 mL) was added to stop the reaction. The resulting solution was washed with 2 N HCl twice (2 × 50 mL), and H_2O twice (2 × 50 mL), passed through a small plug of silica gel, and dried over anhydrous Na_2SO_4. The solvent was removed under reduced pressure to give the product as a colorless liquid (13.0 g, 94% yield).

Surface Hydrosilylation and Introduction of Surface Initiator.[13] A freshly etched silicon chip (10×10 or 20×20 mm^2) was transferred into a vial (80 mL) containing 10 mL neat 10-undecen-1-yl-2-bromo-2-methylpropionate. The bottle was purged with N_2 for 15 min so as to vent the air. The reaction was performed in a CEM Discovery microwave reactor, controlled with a dynamic mode to reach 125 ℃ in 10 min and held there for 20 min. After reaction, the chip was washed ultrasonically with CH_2Cl_2, anhydrous alcohol, and water sequentially, each with 60 mL solution for 3 min respectively, and dried with a mild nitrogen stream.[14-19]

Fabrication of Polymer Brushes

Preparation of PMAA Brushes. PMAA brushes covalently linked to an initiator-grafted silicon chip can be prepared through ATRP of sodium methacrylate, followed by acidification. Surface-initiated polymerization of sodium methacrylate was performed in a N_2-filled vessel with 540 mg sodium methacrylate and 75 μL PMDETA, dissolved in a 6 mL solution of 1:1 (v/v) H_2O/CH_3OH. The bottle was purged with N_2 for 15 min so as to vent the air, followed by addition of 18 mg CuBr, then held at 40 ℃ for 2-4 h. Then, the chips were removed from the vial, washed with THF, H_2O and ethanol sequentially, and dried under a stream of N_2.[14-16,19]

Preparation of PHEMA Brushes. Surface-initiated polymerization of 2-hydroxyethyl methacrylate (HEMA) was performed in a N_2-filled vessel with 3 mg $CuBr_2$ and 75 μL PMDETA dissolved in a 4.5 mL solution of 1:1:1 (v/v/v) H_2O/THF/HEMA. The bottle was

purged with N_2 for 15 min so as to vent the air, followed by addition of 30 mg CuBr, then held at 40 ℃ for 2-4 h. After reaction, the chips were removed from the vials, washed with THF, H_2O and ethanol sequentially, and dried under a stream of N_2.[15,16]

Preparation of PNIPAM Brushes. Surface-initiated polymerization of N-isopropylacrylamide (NIPAM) was performed in a N_2-filled vessel with 560 mg NIPAM and 75 μL PMDETA dissolved in a 6 mL solution of 1:1 H_2O/CH_3OH. The bottle was purged with N_2 for 15 min so as to vent the air, followed by addition of 18 mg CuBr, then held at 40 ℃ for 2-4 h. After reaction, the chips were removed from the vials, washed with THF, H_2O and ethanol sequentially, and dried under a stream of N_2.[14-16,19]

MTR-IR Spectrometry.[20] A Bruker V80 spectrometer equipped our home-made MTR-IR setup was used to illuminate the stepwise reactions. The double-side polished chips (20×20 mm^2) were used as the standard samples for measurements. In all cases, samples were mounted in a vacuum chamber and thus the interference of CO_2 and water vapor from air was greatly attenuated. Spectra were recorded with 100 scans at a 7.5 kHz velocity and a resolution of 4 cm^{-1}, using a cleaned planar Si (100) chip as reference. All spectra were disposed with the OPUS software and spectral windows were zoomed to highlight the significant changes.[9]

Atomic Force Microscopy. The nanostructures on silicon generated in each step were characterized by Dimension FastScan AFM (Bruker, Inc.). All samples were scanned by FastScan A tips in air in ScanAsyst mode except for the pH-responsive studies. The pH-responsive AFM images in aqueous media were scanned by SCANASYST-FLUID+ tips in ScanAsyst mode. Generally, 90 μL hydrochloric acid (0.001 mol/L) at pH 3.0 was dropped on a PMAA nanodot chip, stayed for 1 h, then the chip was imaged; after AFM imaging at pH 3.0, keeping the imaging position without any movement, while 9.1 μL of 0.01 mol/L NaOH was injected into the aqueous drop with microsyringe and the drop stayed for 30 min for diffusion and PMAA expansion equilibrium, then the same region was imaged again by the same AFM tip. All AFM images were disposed with the Nanoscope Analysis software and the statistical analyses were performed using its **Particle Analysis**, **Depth** and **Section** commands.

2. MTR-IR spectra

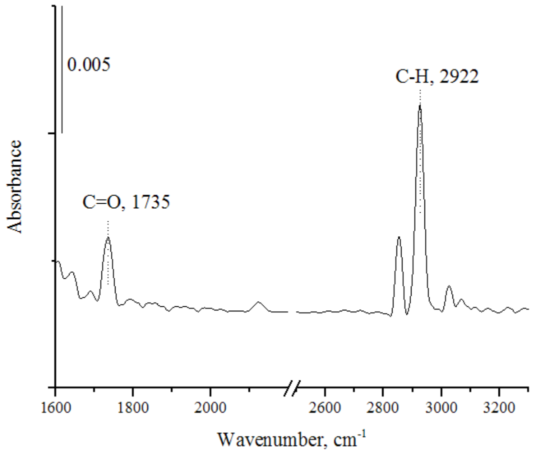

Figure S1. MTR-IR spectrum of the surface initiator monolayer as shown in Fig. 1C.

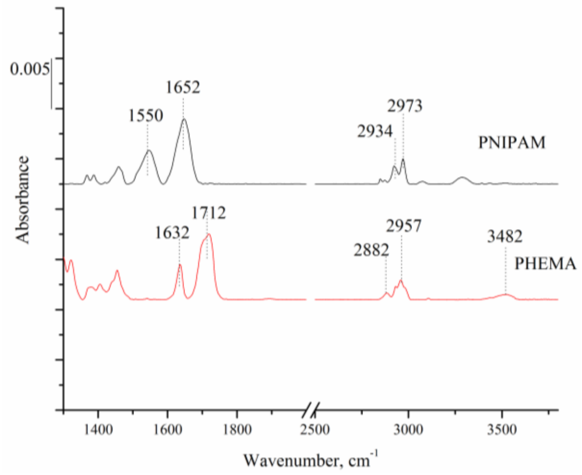

Figure S2. MTR-IR spectra of PNIPAM and PHEMA nanodots.

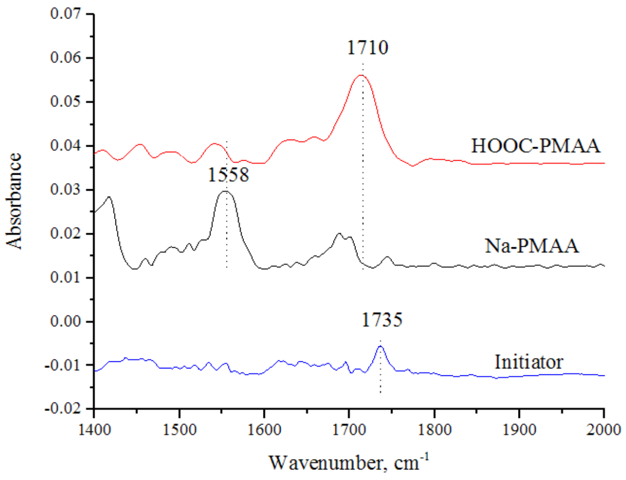

Figure S3. MTR-IR spectra: α-bromoisobutyrate-ended initiator monolayer (Initiator) in Fig. 6F, nano-fingerprints of sodium polymethacrylate (Na-PMAA) and acidified PMAA (HOOC-PMAA) in Fig. 6G.

3. More AFM Images of PMAA Nanodot Arrays

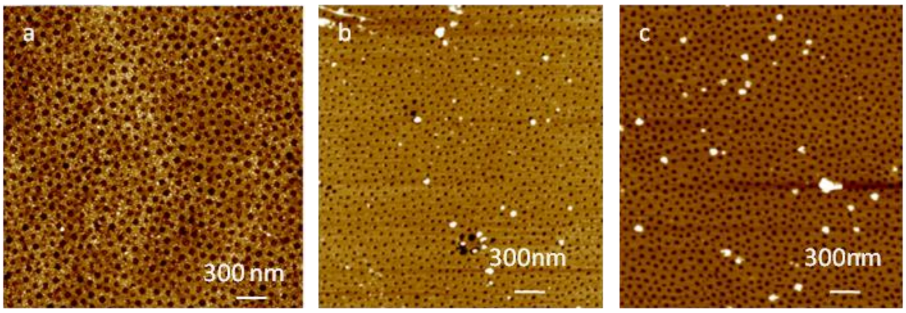

Figure S4a. Non-polymerization. Very few polymers grew from the nanopits.

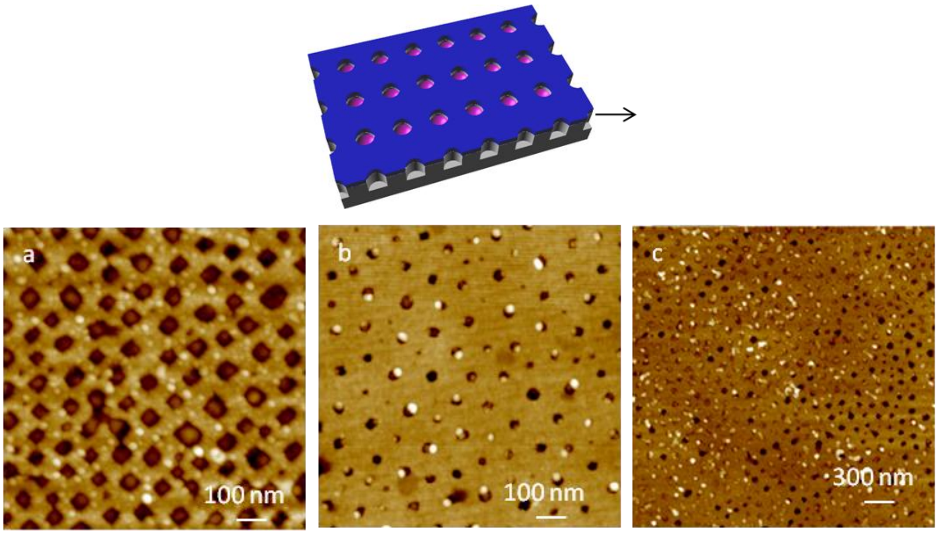

Figure S4b. Off-polymerization: partly-filled nanopits with polymers. Most of the polymer dots are under the silicon surface horizon. Upper, schematic drawing of off-polymerization; lower, real AFM images.

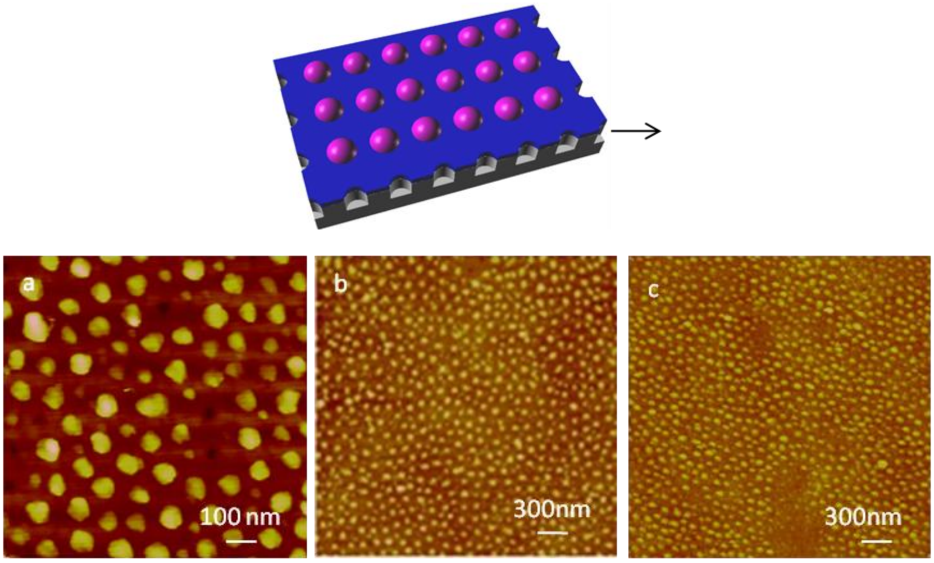

Figure S4c. Proper-polymerization: fully-filled nanopits with polymer nanodots protruding out of the silicon surface horizon. Upper, schematic drawing of proper-polymerization; lower, real AFM images.

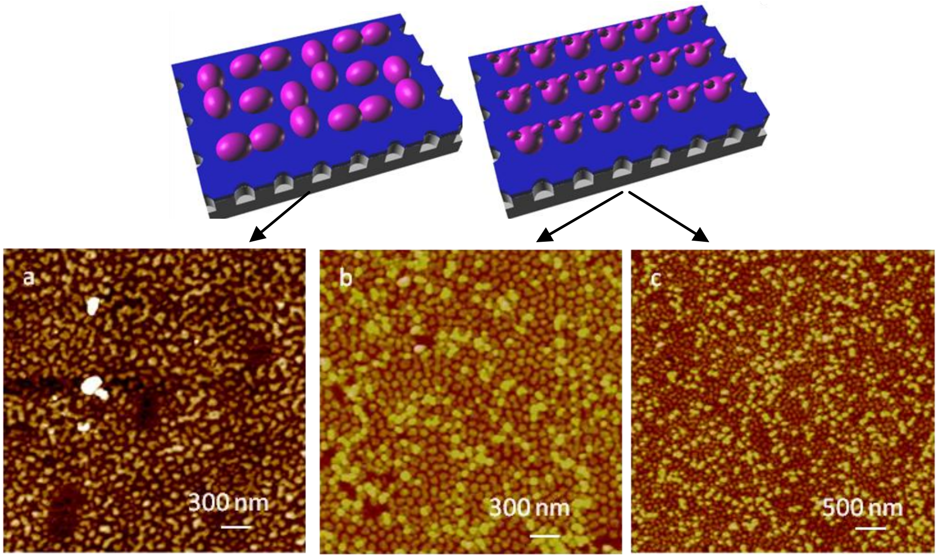

Figure S4d. Over-polymerization: irregular polymer nanodots by over-polymerization including two-layer nanodots. Upper, schematic drawing of over-polymerization; lower, real AFM images.

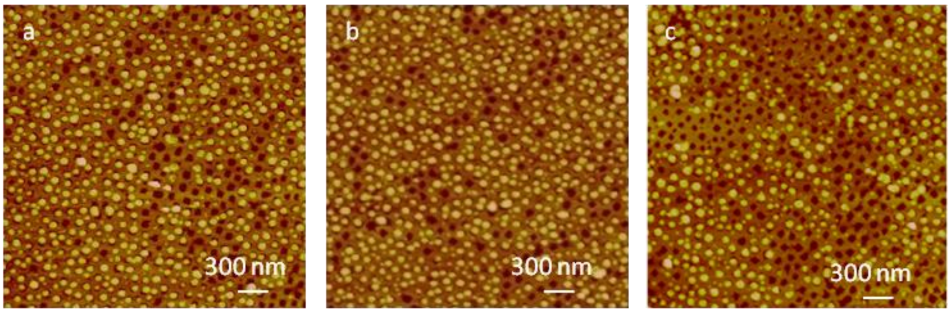

Figure S4e. Mixed nanodots and nanopits: both nanodots and nanopits coexisted in the same chip.

4. Additional pH-Responsive Pair of PMAA Nanodots

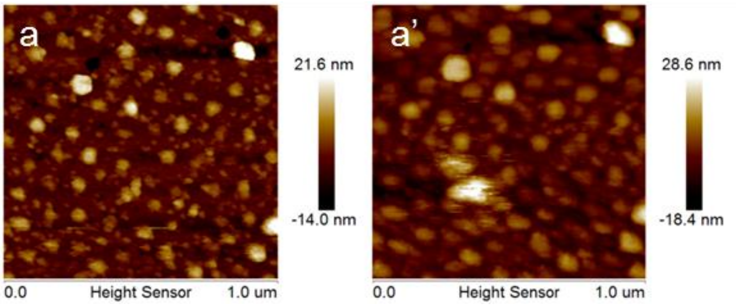

Figure S5. A pair of fluidic AFM images at the same position: the pH-response of PMAA nanodots from pH 3.0 (a) to 9.0 (a') indicates the expansion of nanodot volume.

5. Statistical Analyses of AFM Images

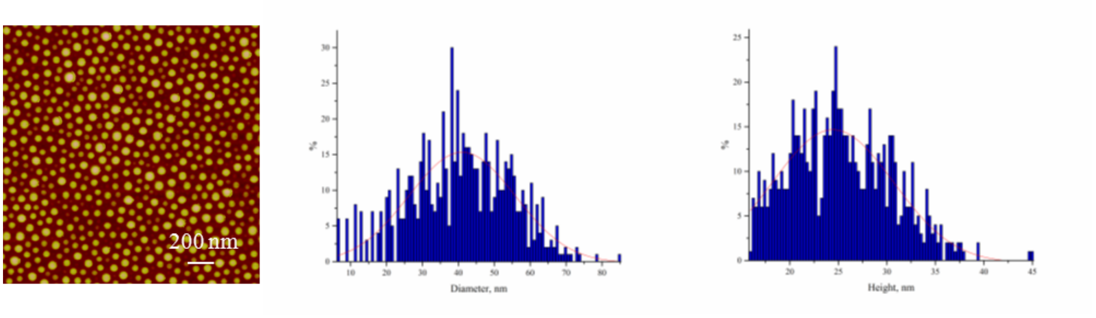

Figure S6. Statistical analyses of Fig. 1A in the main text: left, AFM image of spin-coated PS-*b*-P4VP (109000-*b*-27000) film; middle and right, its statistical analyses of the dot diameter and height in Gauss distribution respectively. A single peak Gaussian function was used to fit each histogram.

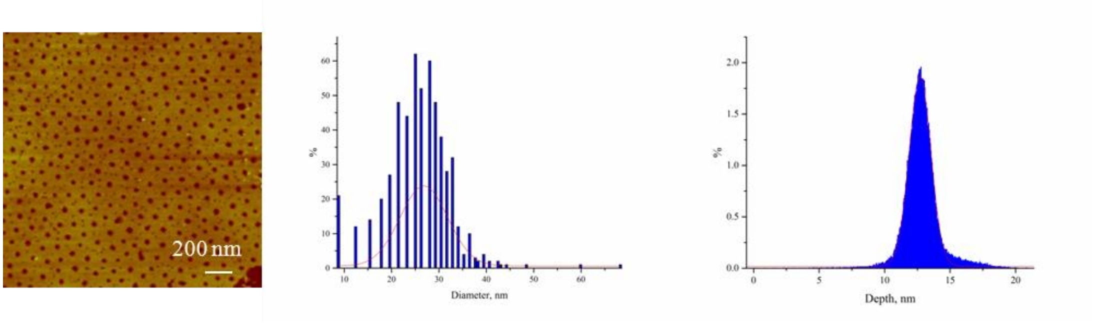

Figure S7. Statistical analyses of Fig. 1B in the main text: left, AFM image of nanopits; middle and right, its statistical analyses of the pit diameter and depth in Gauss distribution respectively. A single peak Gaussian function was used to fit each histogram.

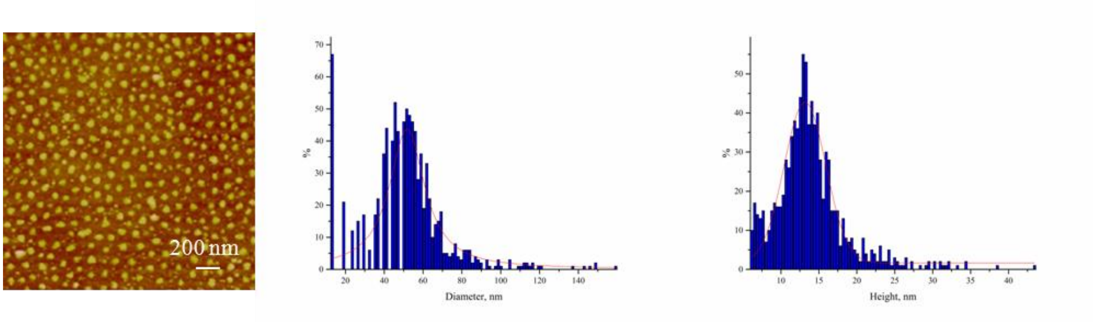

Figure S8. Statistical analyses of Figure 1D in the main text: left, AFM image of PMAA brush nanodots; middle and right, its statistical analyses of the dot diameter and height in Gauss distribution respectively. A single peak Gaussian function was used to fit each histogram.

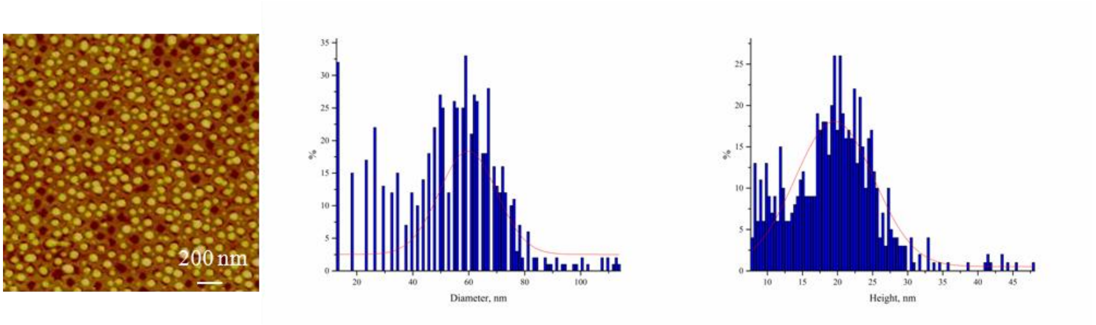

Figure S9. Statistical analyses of Figure 2a in the main text: left, AFM image of “defected” PMAA brush nanodots; middle and right, its statistical analyses of the dot diameter and height in Gauss distribution respectively. A single peak Gaussian function was used to fit each histogram.

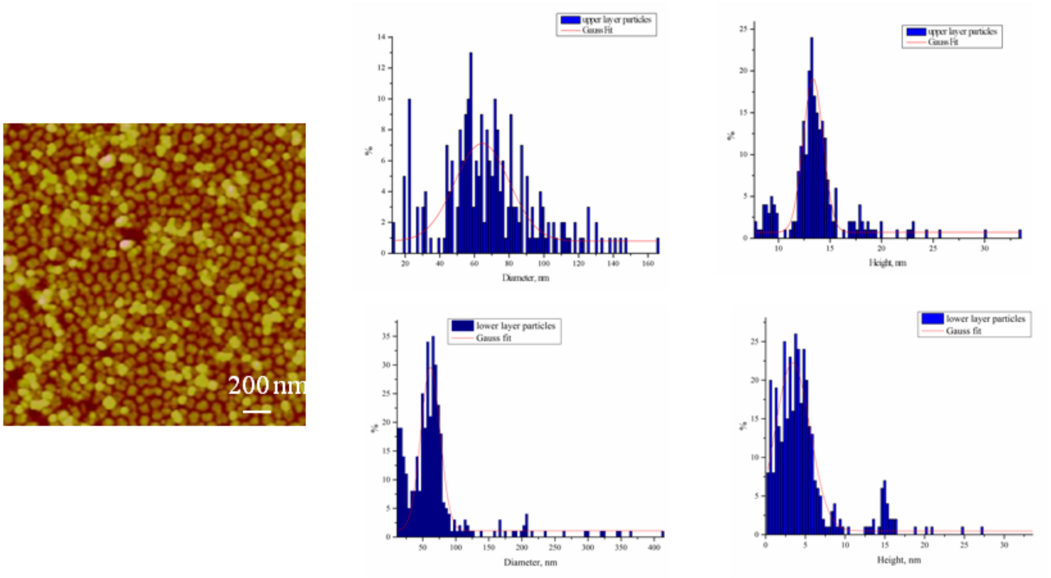

Figure S10. Statistical analyses of Figure 2b in the main text: left, AFM image of “defected” PMAA brush nanodots; upper middle and right, its statistical analyses of the upper-layer dot diameter and height in Gauss distribution respectively; lower middle and right, its statistical analyses of the lower-layer dot diameter and height in Gauss distribution respectively. A single peak Gaussian function was used to fit each histogram.

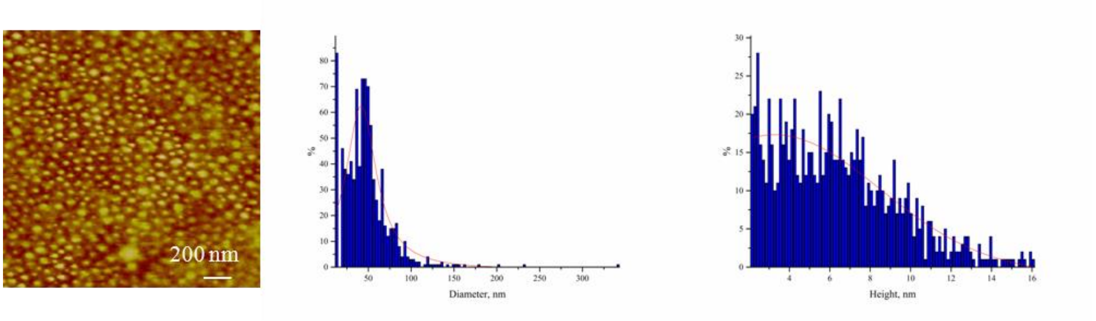

Figure S11. Statistical analyses of an AFM image with PHEMA brush nanodots: left, AFM image of PHEMA brush nanodots; middle and right, its statistical analyses of the dot diameter and height in

Gauss distribution respectively. A single peak Gaussian function was used to fit each histogram.

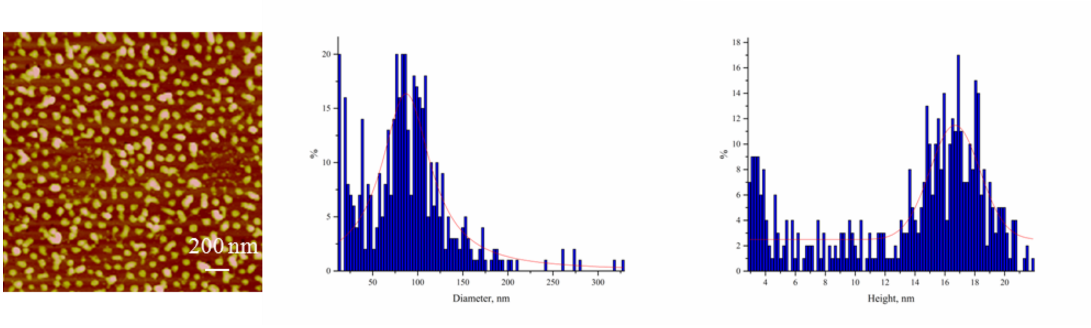

Figure S12. Statistical analyses of an AFM image with PNIPAM brush nanodots: left, AFM image of PNIPAM brush nanodots; middle and right, its statistical analyses of the dot diameter and height in Gauss distribution respectively. A single peak Gaussian function was used to fit each histogram.

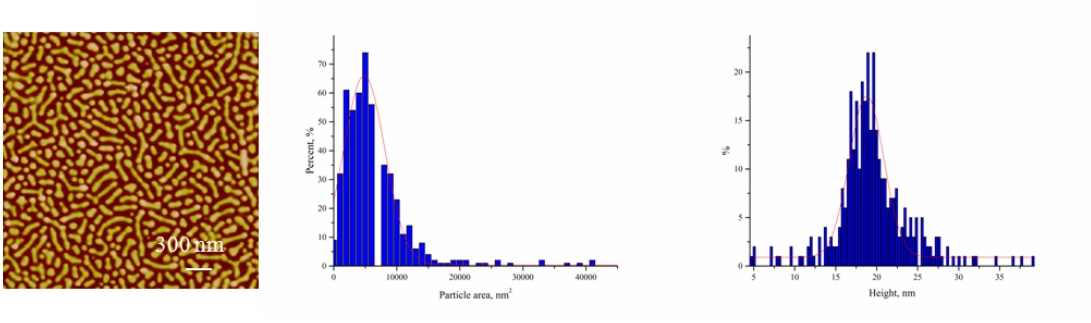

Figure S13. Statistical analyses of Figure 5a in the main text: left, AFM image of PS-*b*-P4VP (330000-*b*-125000) array after spin-coating; middle and right, its satistical analyses of the rod area and height distribution respectively. A single peak Gaussian function was used to fit each histogram.

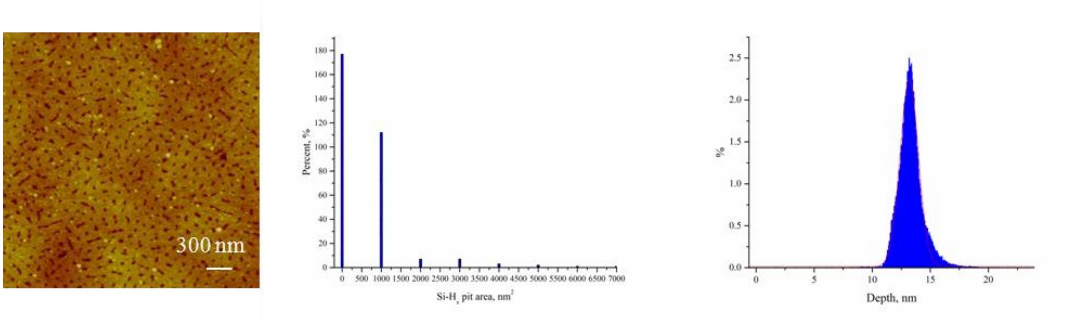

Figure S14. Statistical analyses of Figure 5b in the main text: left, left, AFM image of wet-etched nanoslit array after relieving PS-*b*-P4VP, middle and right, its satistical analyses of the slit area and depth distribution respectively. A single peak Gaussian function was used to fit each histogram.

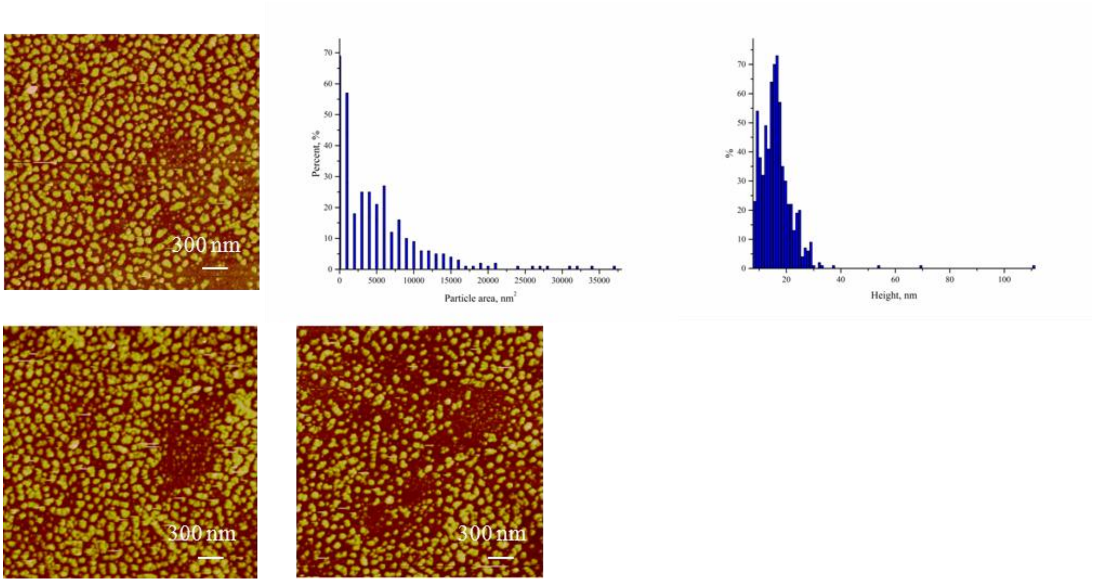

Figure S15. **Statistical analyses of Figure 5c in the main text**: Upper: left, AFM image of PMAA brushes rods of Figure 5c in the main text; middle and right, its satistical analyses of the rod area and height distribution respectively. A single peak Gaussian function was used to fit each histogram. Bottom: additional two AFM images of rod-like arrays.

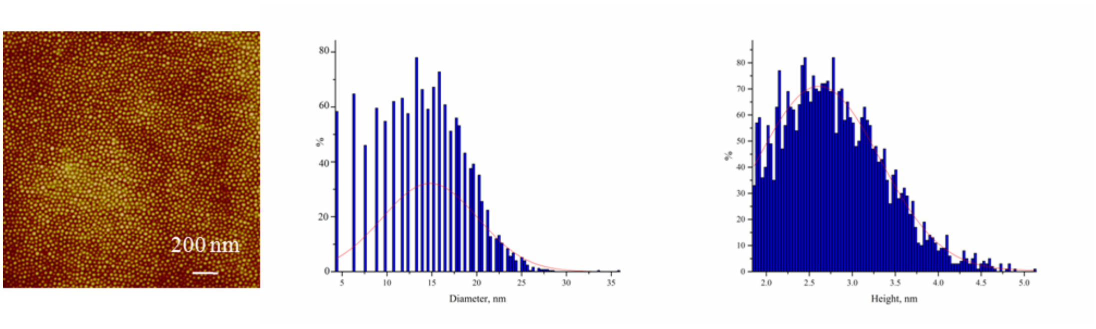

Figure S16. **Statistical analyses of Fig. 6A in the main text:** left, AFM image of spin-coated PS-*b*-P2VP (56000-*b*-21000) film; middle and right, its statistical analyses of the dot diameter and height in Gauss distribution respectively. A single peak Gaussian function was used to fit each histogram.

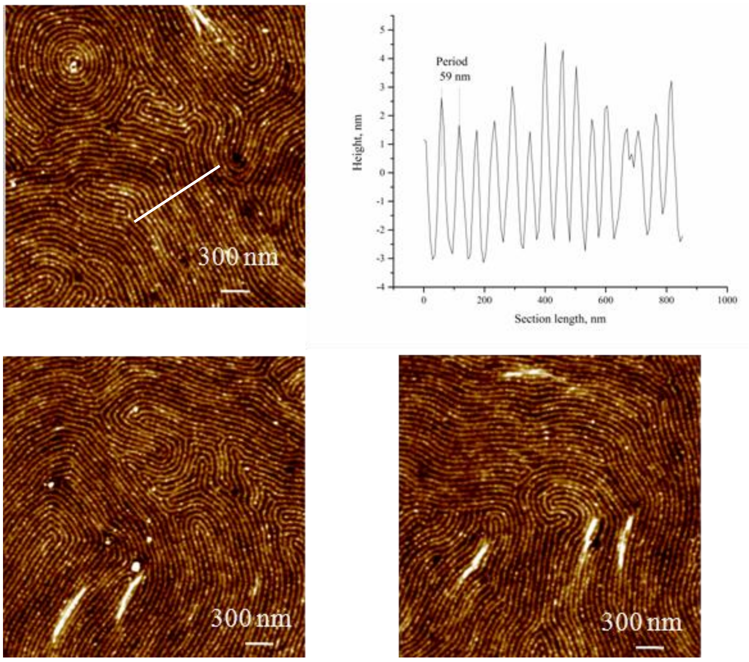

Figure S17. **Cross sectional profile of Figure 6B in the main text**. Upper: left, AFM image of solvent vapor annealed PS-*b*-P2VP fingerprints of Figure 6B in the main text; right, its cross sectional profile. Bottom: additional two AFM images of solvent vapor annealed PS-*b*-P2VP fingerprints.

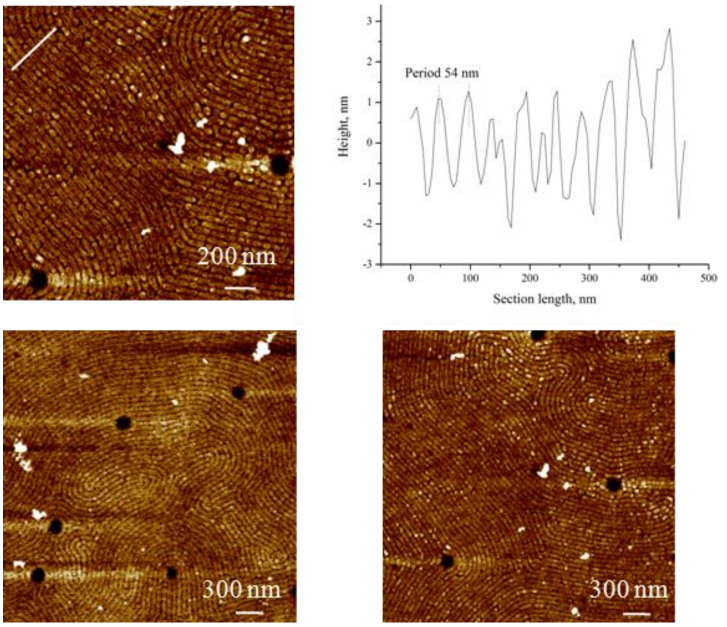

Figure S18. **Cross sectional profile of Figure 6C in the main text**. Upper: left, AFM image of platinichloride deposited PS-*b*-P2VP fingerprints of Figure 6C in the main text; right, its cross sectional profile. Bottom: additional two AFM images of platinichloride deposited PS-*b*-P2VP fingerprints.

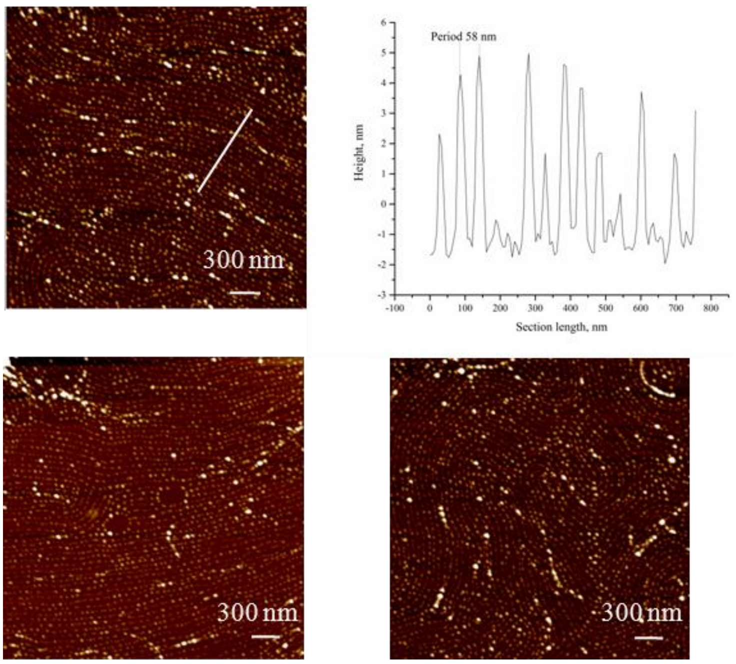

Figure S19. **Cross sectional profile of Figure 6D in the main text**. Upper: left, AFM image of Figure 6D in the main text, platinum dotted fingerprints after O_2 plasma; right, its cross sectional profile. Bottom: additional two AFM images of platinum dotted fingerprints.

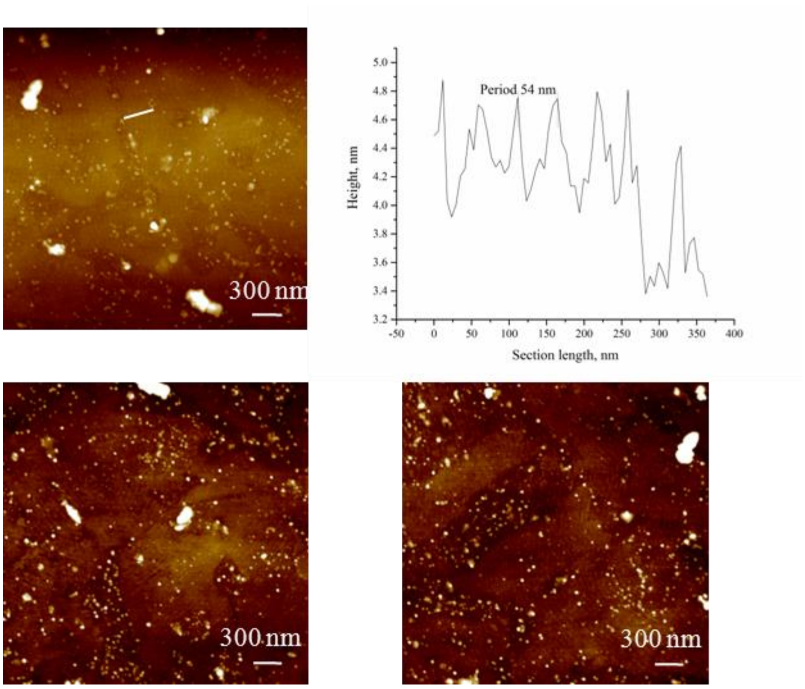

Figure S20. **Cross sectional profile of Figure 6E in the main text**. Upper: left, AFM image of Figure 6E in the main text, SiH*x* pendant fingerprints after etching in a HF/H_2O_2/EtOH (v/v/v = 1:1:3) solution for 3-6 min; right, its cross sectional profile. Bottom: additional two AFM images of fingerprints by metal-assisted chemical etching.

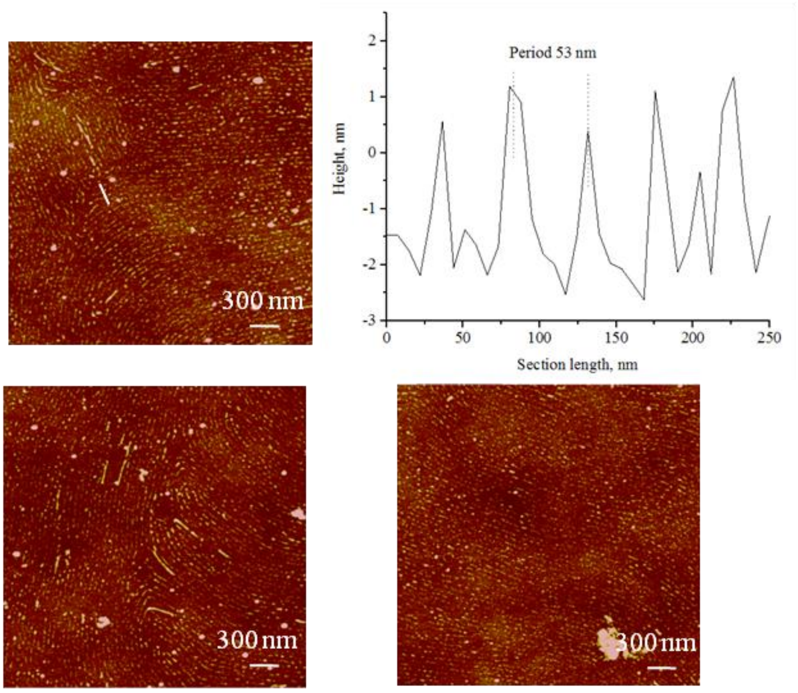

Figure S21. **Cross sectional profile of Figure 6F in the main text**. Upper: left, AFM image of Figure 6F in the main text, initiator fingerprints; right, its cross sectional profile. Bottom: additional two AFM images of initiator-grafted fingerprints.

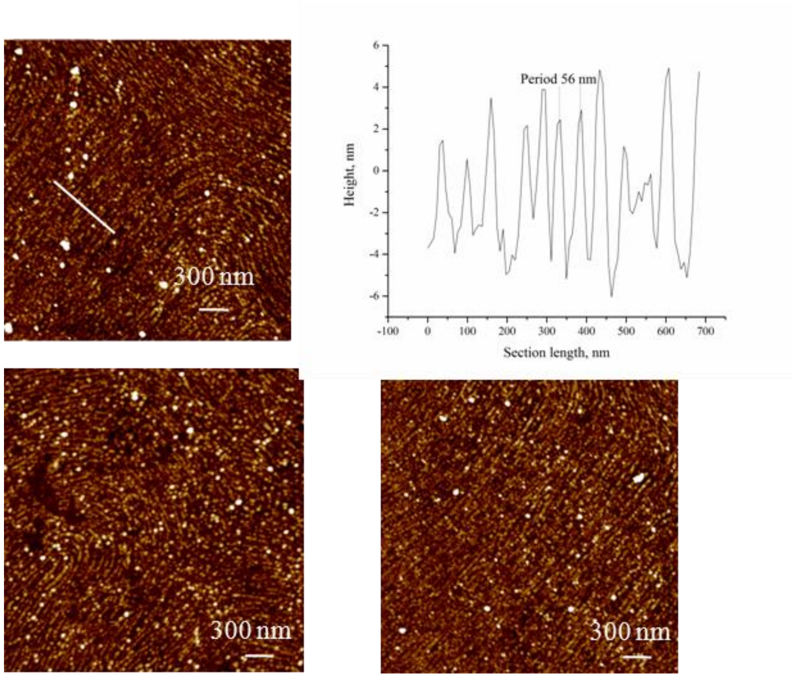

Figure S22. **Cross sectional profile of Figure 6G in the main text**. Upper: left, AFM image of Figure 6G in the main text, PMAA brush fingerprints; right, its cross sectional profile. Bottom: additional two AFM images of PMAA brush fingerprints.

References

1. Wu, N. L. Y., Zhang, X., Murphy, J. N. *et al*. Density doubling of block copolymer templated features. *Nano Lett.* **12**, 264-268 (2012).

2. Deng, X., Buriak, J. M., Dai, P. *et al*. Block copolymer-templated chemical nanopatterning on pyrolyzed photoresist carbon films. *Chem. Commun.* **48**, 9741-9743 (2012).

3. Chai, J., Wang, D., Fan, X. *et al*. Assembly of aligned linear metallic patterns on silicon. *Nat. Nanotechnol.* **2**, 500-506 (2007).

4. Chai, J., Buriak, J. M. Using cylindrical domains of block copolymers to self-assemble and align metallic nanowires. *ACS Nano* **2**, 489-501 (2008).

5. Zhang, X., Qiao, Y., Xu, L. *et al*. Constructing metal-based structures on nanopatterned etched silicon. *ACS Nano* **5**, 5015-5024 (2011).

6. Qiao, Y., Wang, D., Buriak, J. M. Block copolymer templated etching on silicon. *Nano lett.* **7**, 464-469 (2007).

7. Aizawa, M., Buriak, J. M. Block copolymer-templated chemistry on Si, Ge, InP, and GaAs surfaces. *J. Am. Chem. Soc.* **127**, 8932-8933 (2005).

8. Zhang, X., Harris, K. D., Wu, N. L. Y. *et al*. Fast assembly of ordered block copolymer nanostructures through microwave annealing. *ACS Nano* **4**, 7021-7029 (2010).

9. Raynor, J. E. Saccharide polymer brushes to control protein and cell adhesion to titanium. *Biomacromolecules* **10**, 748-755 (2009).

10. Sanjuan, S., Tran, Y. Synthesis of random polyampholyte brushes by atom transfer radical polymerization. *J. Polym. Sci. Part A: Polym. Chem.* **46**, 4305-4319 (2008).

11. Matyjaszewski, K., Miller, P. J., Shukla, N. *et al*. Polymers at interfaces: using atom transfer radical polymerization in the controlled growth of homopolymers and block copolymers from silicon surfaces in the absence of untethered sacrificial initiator. *Macromolecules* **32**, 8716-8724 (1999).

12. Jia, Z., Yuan, W., Zhao, H. *et al*. Composite electrolytes comprised of poly (ethylene oxide) and silica nanoparticles with grafted poly (ethylene oxide)-containing polymers. *RSC Adv.* **4**, 41087-41098 (2014).

13. Buriak, J. M. Illuminating silicon surface hydrosilylation: an unexpected plurality of mechanisms. *Chem. Mater.* **26**, 763-772 (2014).

14. Wang, C., Yan, Q., Liu, H. *et al*. Different EDC/NHS activation mechanisms between PAA and PMAA brushes and the following amidation reactions. *Langmuir* **27**, 12058-12068 (2011).

15. Tugulu, S., Barbey, R., Harms, M. *et al*. Synthesis of poly (methacrylic acid) brushes via surface-initiated atom

transfer radical polymerization of sodium methacrylate and their use as substrates for the mineralization of calcium carbonate. *Macromolecules* **40**, 168-177 (2007).

16. Linford, M. R., Fenter, P., Eisenberger, P. M. *et al*. Alkyl monolayers on silicon prepared from 1-alkenes and hydrogen-terminated silicon. *J. Am. Chem. Soc.* **117**, 3145-3155 (1995).

17. Hurley, P. T., Ribbe, A. E., Buriak, J. M. Nanopatterning of alkynes on hydrogen-terminated silicon surfaces by scanning probe-induced cathodic electrografting. *J. Am. Chem. Soc.* **125**, 11334-11339 (2003).

18. Sieval, A. B., Vleeming, V., Zuilhof, H. *et al*. Monolayers of 1-alkynes on the H-terminated Si (100) surface. *Langmuir* **15**, 8288-8291 (1999).

19. Munirasu, S., Karunakaran, R. G., Rühe, J. *et al.* Synthesis and morphological study of thick benzyl methacrylate–styrene diblock copolymer brushes. *Langmuir* **27**, 13284-13292 (2011).

20. Liu, H., Venkataraman, N. V., Bauert, T. E. *et al.* Multiple transmission−reflection infrared spectroscopy for high-sensitivity measurement of molecular monolayers on silicon surfaces. *Phys. Chem. A* **112**, 12372-12377 (2008).
